# Enrichment post-library preparation enhances the sensitivity of high-throughput sequencing-based detection and characterization of viruses from complex samples

**DOI:** 10.1186/s12864-019-5543-2

**Published:** 2019-02-26

**Authors:** Adrian C. Paskey, Kenneth G. Frey, Gary Schroth, Stephen Gross, Theron Hamilton, Kimberly A. Bishop-Lilly

**Affiliations:** 1 Genomics and Bioinformatics Department, Biological Defense Research Directorate, Naval Medical Research Center – Frederick, Fort Detrick, Frederick, MD 21702 USA; 2 Uniformed Services University of the Health Sciences, Bethesda, MD 20814 USA; 3 Leidos, Reston, VA 20190 USA; 4 Illumina Inc., San Diego, CA 92122 USA

**Keywords:** Genome enrichment, Virus detection, High-throughput sequencing, Virome profiling, Virome capture sequencing, Viral enrichment, Sequencing-based detection, Next generation sequencing, Hybridization-based target enrichment

## Abstract

**Background:**

Sequencing-based detection and characterization of viruses in complex samples can suffer from lack of sensitivity due to a variety of factors including, but not limited to, low titer, small genome size, and contribution of host or environmental nucleic acids. Hybridization-based target enrichment is one potential method for increasing the sensitivity of viral detection via high-throughput sequencing.

**Results:**

This study expands upon two previously developed panels of virus enrichment probes (for filoviruses and for respiratory viruses) to include other viruses of biodefense and/or biosurveillance concern to the U.S. Department of Defense and various international public health agencies. The newly expanded and combined panel is tested using carefully constructed synthetic metagenomic samples that contain clinically relevant amounts of viral genetic material. Target enrichment results in a dramatic increase in sensitivity for virus detection as compared to shotgun sequencing, yielding full, deeply covered viral genomes from materials with Ct values suggesting that amplicon sequencing would be likely to fail. Increased pooling to improve cost- and time-effectiveness does not negatively affect the ability to obtain full-length viral genomes, even in the case of co-infections, although as expected, it does decrease depth of coverage.

**Conclusions:**

Hybridization-based target enrichment is an effective solution to obtain full-length viral genomes for samples from which virus detection would fail via unbiased, shotgun sequencing or even via amplicon sequencing. As the development and testing of probe sets for viral target enrichment expands and continues, the application of this technique, in conjunction with deeper pooling strategies, could make high-throughput sequencing more economical for routine use in biosurveillance, biodefense and outbreak investigations.

**Electronic supplementary material:**

The online version of this article (10.1186/s12864-019-5543-2) contains supplementary material, which is available to authorized users.

## Background

High-Throughput Sequencing (HTS), also known as Next-Generation Sequencing (NGS), has many advantages for pathogen detection as compared to traditional methods such as Polymerase Chain Reaction (PCR), serological assays, and/or culture-based methods. Metagenomic sequencing is the high-throughput sequencing of nucleic acid from complex samples rather than from purified microorganisms. Metagenomic sequencing is much less biased than other methods and allows for the detection of fastidious or nonculturable organisms as well as multiple unrelated pathogens within a single sample [1]. Moreover, detection via HTS is much less susceptible to false-negative results caused by antigenic drift or signature erosion. Despite these advantages, one of the technical challenges encountered with respect to metagenomic sequencing is obtaining adequate depth and breadth of coverage from pathogens like RNA viruses that i) typically have small genomes, and ii) are typically present at low titers amidst the background ‘noise’ of the host and commensals [1]. Genome size directly affects sensitivity of detection by HTS because the sampling of sequence fragments within a sample depends on the prevalence of those fragments and organisms with larger genomes typically contribute more fragments, therefore being sampled more often than organisms with smaller genomes. In other words, organisms with larger genomes have the potential to contribute a larger proportion to the overall number of sequencing reads even when the plaque-forming units (PFU), or colony-forming units (CFU) in the case of bacteria, are equivalent to that of an organism with a smaller sized genome.

Although conventional shotgun sequencing allows for the detection of all domains of life, it rarely returns robust coverage of a small viral genome when taken from a very complex sample. A variety of possible strategies exist to enhance the sensitivity of HTS for virus detection and characterization, including purification of specific viral fractions by physical methods such as filtration and ultracentrifugation [2], amplicon-based target enrichment, and hybridization-based target enrichment. Purification of viral fractions is ideal in some cases, although it can be laborious, and for certain sized samples (for instance clinical samples of very limited volume) it may not be realistic. The use of hybridization-based target enrichment could be preferable to the aforementioned technologies because it has the potential to yield sequence data covering the entire genome of multiple viruses with just one sequencing reaction by using genome-wide probes designed against multiple viruses to specifically select for viral cDNA prior to sequencing. Amplicon sequencing of viral genomes is a technique that has been widely used, but it has some disadvantages, which were articulated by Metsky et al. in a recent study of Zika virus (ZIKV) [3]. First, traditional amplicon sequencing typically requires technically challenging normalization and pooling of individual amplicons to cover the entire genome of one specific virus. However, recently a protocol was published for efficient amplicon primer design and multiplex amplicon generation in a single tube for sequencing in the MinION or Illumina platforms [4]. Although this method obviates the amplicon normalization and pooling steps and is effective for producing whole genome sequence data from a low titer ZIKV sample, this method has not been demonstrated for production of whole genome sequence data for multiple diverse viruses from a single complex sample. Additionally, amplicon sequencing typically requires as much as 40 cycles of PCR amplification [4–6], which can introduce sequence errors. Furthermore, amplicon-based sequencing is vulnerable to false negative results caused by mutations in primer binding sites, as was recently demonstrated for Dengue virus (DENV) [5] as well as false positive variant results possibly caused by low and/or uneven coverage [7]. By contrast, the use of probes tiled along the entire length of a viral genome to hybridize and select for virus-specific fragments has the potential to produce less false-negative pathogen detection results by virtue of many more potential binding sites along an individual genome and resulting more uniform coverage.

Ebola virus (EBOV) is one specific example of a pathogen for which false-negative PCR results can have devastating consequences and for which available PCR-based assay effectiveness has been shown to be affected by drift [8]. Therefore, a panel of 80-mer oligonucleotide probes designed against eight Filovirus genomes was recently used for post-sequencing library enrichment in a HTS-based study of a recent EBOV outbreak in West Africa and in an investigation of potential genetic variation of EBOV in experimentally infected nonhuman primates [9–11]. In this protocol, viral enrichment is coupled with the RNA Access kit, developed by Illumina, Inc. The technical advancements of the RNA Access kit had already enabled the sequencing of previously unsequencable materials such as those of low concentration and formalin-fixed paraffin-embedded (FFPE) tissue [12], and now this protocol has been employed not only for the detection and characterization of EBOV from clinical samples but also for detection and characterization of respiratory viruses in clinical samples [13, 14]. In general, hybridization-based viral target enrichment has been successfully employed to characterize viruses found within both contrived samples and clinical samples [3, 9, 10, 13, 15–20]. The performance of the Respiratory Virus Panel (RVP) version of this method [14], which uses probes for 34 common respiratory viruses in conjunction with the TruSeq RNA Access protocol, was recently investigated and it was demonstrated to work well overall when tested on human clinical samples [13]. Specifically, the authors reported successful enrichment for 30 of 33 human clinical samples tested. Importantly, RT-PCR was conducted on those same samples and Ct values of respiratory viruses in those clinical samples ranged from 21 to 33 [13], which provides a framework for beginning to assess the limits of detection of hybridization-based enrichment sequencing. Herein, we extend this approach by i) expanding this viral probe panel to include viruses of biosurveillance and biodefense concern and ii) employing carefully constructed mock clinical samples to systematically assess this technique’s performance in a variety of conditions, such as deeper multiplexing for cost effectiveness as well as more extensive co-infection scenarios. We demonstrate the sensitivity and reproducibility of hybridization-based viral enrichment sequencing despite virus divergence and we show that this sensitivity is maintained even with extensive multiplexing of samples to decrease cost. Herein we demonstrate that within one reaction tube, this technique can even be used to detect and discriminate between multiple serotypes of a virus within a clinical sample or to detect and discriminate amongst multiple unrelated viruses that present similar clinical symptoms, and we demonstrate this performance at clinically relevant concentrations of virus.

## Results

### Hybridization-based target enrichment enhances sensitivity of HTS for detection of virus in complex environmental samples

Enhanced detection of viruses from various clinical sample types using filovirus- or respiratory virus-specific probes has recently been demonstrated [9, 10, 13]. To expand the range of viruses that could be detected, in this study those two probe panels were combined with new probes for 41 additional viruses that are of biosurveillance and biodefense concern, for a full panel targeting 83 diverse viruses (Table 1). In order to test this newly expanded probe panel and to specifically assess the effect of hybridization-based viral enrichment on the sensitivity of HTS for detection of a single virus within a complex environmental sample, commercial bat guano was spiked with increasing concentrations of Influenza virus (IFV). Spiked samples were split into two parts each, with each part being processed in parallel with unbiased, shotgun sequencing versus target enrichment sequencing using an expanded panel of probes.

**Table 1:** Viruses included in target enrichment panel

Virus	Genome size (kb)	Genome type	NCBI accession(s) of reference used in probe design	Notes
Nipah virus	18,246	Negative sense ssRNA	NC_002728.1	New addition to probe panel
Bat Paramyxovirus	18,530	Negative sense ssRNA	NC_025256.1	New addition to probe panel
Cedar virus	18,162	Negative sense ssRNA	JQ001776.1	New addition to probe panel
Hendra virus	18,234	Negative sense ssRNA	NC_001906.3	New addition to probe panel
Tioman virus	15,522	Negative sense ssRNA	NC_004074.1	New addition to probe panel
Menangle virus	15,516	Negative sense ssRNA	NC_007620.1	New addition to probe panel
Middle East Respiratory Syndrome Coronavirus	30,094	Positive sense ssRNA	KJ614529.1	New addition to probe panel
Severe Acute Respiratory Syndrome virus	29,751	Positive sense ssRNA	NC_004718.3	New addition to probe panel
Lujo virus	10,352	Negative sense ssRNA	NC_012776.1, NC_012777.1	New addition to probe panel
Lassa fever virus	10,681	Negative sense ssRNA	NC_004296.1, NC_004297.1	New addition to probe panel
Machupo virus	10,635	Negative sense ssRNA	NC_005078.1, NC_005079.1	New addition to probe panel
Junin virus	10,525	Negative sense ssRNA	NC_005080.1, NC_005081.1	New addition to probe panel
Guanarito virus	10,424	Negative sense ssRNA	NC_005077.1, NC_005082.1	New addition to probe panel
Chapare virus	10,464	Negative sense ssRNA	NC_010562.1, NC_010563.1	New addition to probe panel
Sabia virus	10,499	Negative sense ssRNA	NC_006313.1, NC_006317.1	New addition to probe panel
Hantaan virus	11,845	Negative sense ssRNA	NC_005218.1, NC_005219.1, NC_005222.1	New addition to probe panel
Puumala virus	12,062	Negative sense ssRNA	NC_005223.1, NC_005224.1, NC_005225.1	New addition to probe panel
Sin nombre virus	12,317	Negative sense ssRNA	NC_005215.1, NC_005216.1, NC_005217.1	New addition to probe panel
Andes virus	12,104	Negative sense ssRNA	NC_003466.1, NC_003467.1, NC_003468.1	New addition to probe panel
Rift Valley fever virus	11,979	Negative sense ssRNA	NC_014395.1, NC_014396.1, NC_014397.1	New addition to probe panel
Crimean Congo hemorrhagic fever virus	19,146	Negative sense ssRNA	NC_005300.2, NC_005301.3, NC_005302.1	New addition to probe panel
Omsk hemorrhagic fever virus	10,787	Positive sense ssRNA	NC_005062.1	New addition to probe panel
Kyasanur forest disease virus	10,774	Positive sense ssRNA	JF416958.1	New addition to probe panel
Alkhurma hemorrhagic fever virus	10,685	Positive sense ssRNA	NC_004355.1	New addition to probe panel
Eastern equine encephalitis virus	11,703	Positive sense ssRNA	KJ469643.1	New addition to probe panel
Dengue type 1 virus	10,721	Positive sense ssRNA	AF309641.1	New addition to probe panel
Dengue type 2 virus	10,723	Positive sense ssRNA	EF051521.1	New addition to probe panel
Dengue type 3 virus	10,707	Positive sense ssRNA	AY662691	New addition to probe panel
Dengue type 4 virus	10,653	Positive sense ssRNA	AY618989	New addition to probe panel
Chikungunya virus	11,826	Positive sense ssRNA	NC_004162	New addition to probe panel
Bat coronavirus CDPHE15	28,035	Positive sense ssRNA	NC_022103.1	New addition to probe panel
Bat coronavirus 1A	28,326	Positive sense ssRNA	NC_010437.1	New addition to probe panel
Bat coronavirus 1B	28,476	Positive sense ssRNA	NC_010436.1	New addition to probe panel
Bat coronavirus HKU2	27,165	Positive sense ssRNA	NC_009988.1	New addition to probe panel
Bat SARS coronavirus HKU3–4	29,704	Positive sense ssRNA	GQ153539.1	New addition to probe panel
Bat coronavirus HKU4	30,286	Positive sense ssRNA	NC_009019	New addition to probe panel
Bat coronavirus HKU5–1	30,482	Positive sense ssRNA	NC_009020	New addition to probe panel
Bat coronavirus HKU8	28,773	Positive sense ssRNA	NC_010438.1	New addition to probe panel
Bat coronavirus HKU9–1	29,114	Positive sense ssRNA	NC_009021.1	New addition to probe panel
Bat coronavirus HKU10	28,494	Positive sense ssRNA	NC_018871.1	New addition to probe panel
Zika virus	10,794	Positive sense ssRNA	NC_012532	New addition to probe panel
Respiratory Syncytial virus B (S2)	15,190	Negative sense ssRNA	NC_001803.1	Previously used in [13, 20]
Respiratory Syncytial virus A	15,225	Negative sense ssRNA	AY353550	Previously used in [13, 20]
Influenza virus A (H9N2)	13,500	Negative sense ssRNA	NC_004905.2, NC_004906.1, NC_004907.1, NC_004908.1, NC_004909.1, NC_004910.1, NC_004911.1, NC_004912.1	Previously used in [13, 20]
Influenza virus A (H2N2)	13,460	Negative sense ssRNA	NC_007374.1, NC_007375.1, NC_007376.1, NC_007377.1, NC_007378.1, NC_007380.1, NC_007381.1, NC_007382.1	Previously used in [13, 20]
Influenza virus A (H3N2)	13,630	Negative sense ssRNA	NC_007366.1, NC_007367.1, NC_007368.1, NC_007369.1, NC_007370.1, NC_007371.1, NC_007372.1, NC_007373.1	Previously used in [13, 20]
Influenza virus A (H1N1)	13,590	Negative sense ssRNA	NC_002016.1, NC_002017.1, NC_002018.1, NC_002019.1, NC_002020.1, NC_002021.1, NC_002022.1, NC_002023.1,	Previously used in [13, 20]
Influenza virus A (H5N1)	13,590	Negative sense ssRNA	NC_007357.1, NC_007358.1, NC_007359.1, NC_007360.1, NC_007361.1, NC_007362.1, NC_007363.1, NC_007364.1	Previously used in [13, 20]
Influenza virus A (H7N9)	13,590	Negative sense ssRNA	KC885955, KC885956, KC885957, KC885958, KC885959, KC885960, KC885961, KC885962	Previously used in [13, 20]
Influenza virus B	14,450	Negative sense ssRNA	NC_002204.1, NC_002205.1, NC_002206.1, NC_002207.1, NC_002208.1, NC_002209.1, NC_002210.1, NC_002211.1	Previously used in [13, 20]
Parainfluenza virus 1	15,600	Negative sense ssRNA	NC_003461.1	Previously used in [13, 20]
Parainfluenza virus 2	15,650	Negative sense ssRNA	NC_003443.1	Previously used in [13, 20]
Parainfluenza virus 3	15,460	Negative sense ssRNA	NC_001796.2	Previously used in [13, 20]
Parainfluenza virus 4	17,050	Negative sense ssRNA	NC_021928.1	Previously used in [13, 20]
Human metapneumovirus	13,340	Negative sense ssRNA	NC_004148.2	Previously used in [13, 20]
Adenovirus C	35,937	dsDNA	NC_001405.1	Previously used in [13, 20]
Adenovirus B	35,343	dsDNA	NC_011203.1	Previously used in [13, 20]
Adenovirus E	35,994	dsDNA	NC_003266.2	Previously used in [13, 20]
Human Coronavirus HKU1	29,930	Positive sense ssRNA	NC_006577.2	Previously used in [13, 20]
Human Coronavirus NL63	27,550	Positive sense ssRNA	NC_005831.2	Previously used in [13, 20]
Human Coronavirus 229E	27,320	Positive sense ssRNA	NC_002645.1	Previously used in [13, 20]
Human Coronavirus OC43	30,738	Positive sense ssRNA	AY391777.1	Previously used in [13, 20]
Rhinovirus A	7150	Positive sense ssRNA	NC_001617.1	Previously used in [13, 20]
Rhinovirus C	7100	Positive sense ssRNA	NC_001490.1	Previously used in [13, 20]
Rhinovirus B14	7210	Positive sense ssRNA	NC_001490.1	Previously used in [13, 20]
Human Bocavirus 1	5299	ssDNA	NC_007455.1	Previously used in [13, 20]
Human Bocavirus 2	5196	ssDNA	NC_012042.1	Previously used in [13, 20]
Human Bocavirus 3	5242	ssDNA	NC_012564.1	Previously used in [13, 20]
Human Bocavirus 4	5104	ssDNA	NC_012729.2	Previously used in [13, 20]
KI polyomavirus	5040	dsDNA	NC_009238.1	Previously used in [13, 20]
WU polyomavirus	5229	dsDNA	NC_009539.1	Previously used in [13, 20]
Human parechovirus type 1	7296	Positive sense ssRNA	FM242866.1	Previously used in [13, 20]
Human parechovirus type 6	7347	Positive sense ssRNA	AB252582.1	Previously used in [13, 20]
Human Enterovirus C104	7408	Positive sense ssRNA	AB686524.1	Previously used in [13, 20]
Human Enterovirus C109	7354	Positive sense ssRNA	GQ865517.1	Previously used in [13, 20]
Lloviu cuevavirus	18,927	Negative sense ssRNA	NC_016144	Previously used in [9–11]
Bundibugyo ebolavirus	18,940	Negative sense ssRNA	NC_014373	Previously used in [9–11]
Zaire ebolavirus	18,959	Negative sense ssRNA	NC_002549	Previously used in [9–11]
Reston ebolavirus	18,891	Negative sense ssRNA	NC_004161	Previously used in [9–11]
Sudan ebolavirus	18,875	Negative sense ssRNA	NC_006432	Previously used in [9–11]
Tai Forest ebolavirus	18,935	Negative sense ssRNA	NC_014372	Previously used in [9–11]
Marburg virus (isolate Marburg virus)	19,111	Negative sense ssRNA	NC_001608	Previously used in [9–11]
Marburg virus (isolate Ravn virus)	19,114	Negative sense ssRNA	NC_024781	Previously used in [9–11]

**Fig. 1:**
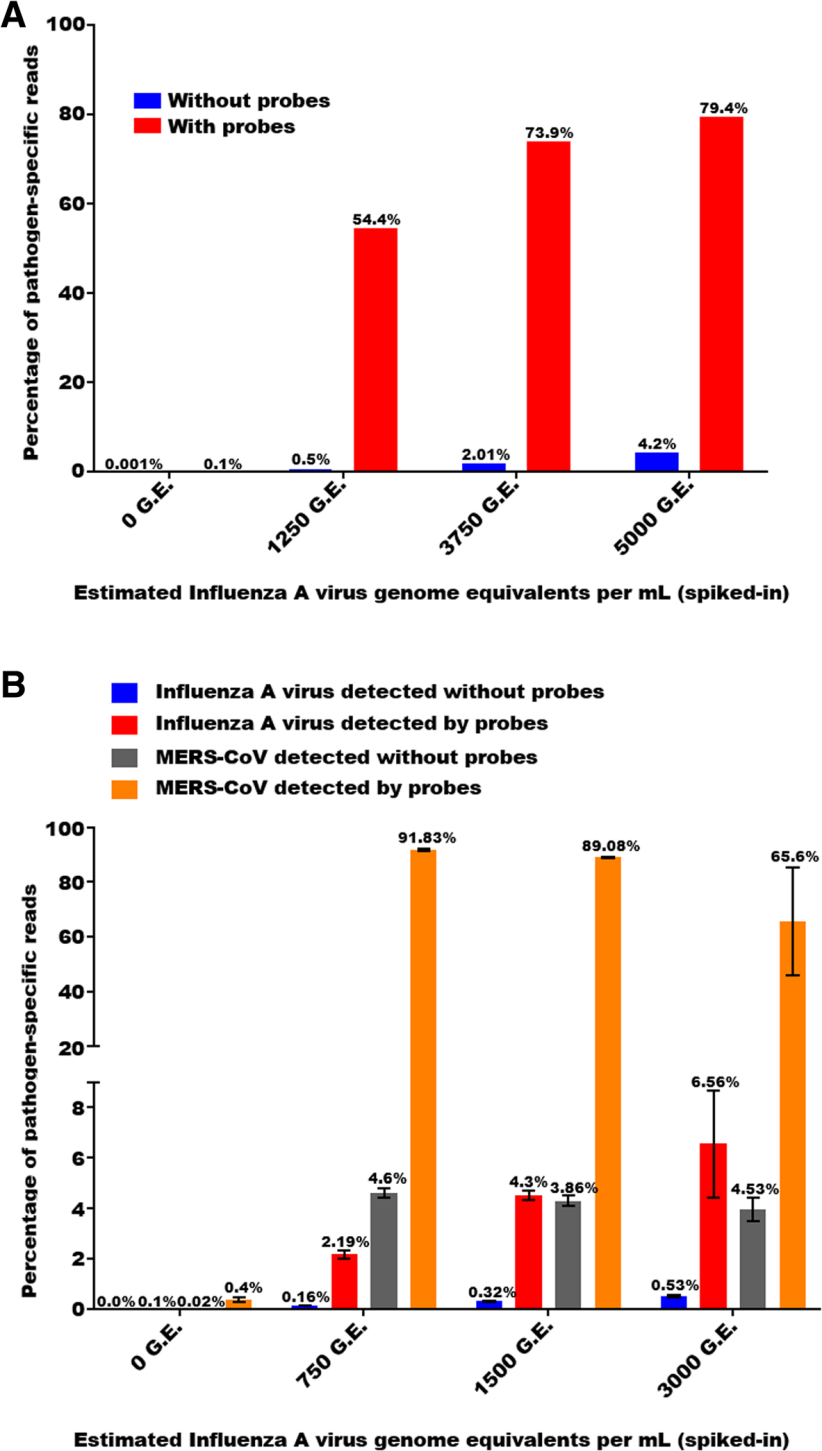
Hybridization-based enrichment enhances sensitivity of HTS for viral detection from complex samples. Known concentrations of IFV genomic RNA (gRNA) were spiked into complex matrices. Samples were split into two parts and processed in parallel via unbiased, shotgun sequencing or target enrichment sequencing in pools of four. **a** IFV was spiked into bat guano at increasing concentrations to simulate environmental-type samples. Shown here is the percentage of IFV-specific reads. **b** Increasing concentrations of IFV gRNA were spiked into total RNA derived from MERS-CoV cell culture lysate. MERS-CoV genomic material was present at a constant, high level amongst all samples. The average percentage of IFV and MERS-CoV virus-specific reads derived from three biological replicates is shown. Black bars denote standard error of the mean for each sample

As expected, a dose-dependent effect in the proportion of sequencing reads derived from IFV was observed as the number of spiked genome copies increased (Fig. 1a and Additional file 1: Table S1), in both the unbiased shotgun sequence data as well as the virus enriched sequence data. However, in this context, hybridization-based target enrichment resulted in approximately 20- to 100-fold more sensitivity for detection of IFV as compared to detection via unbiased, shotgun sequencing. At the lowest concentration tested (1250 genome equivalents (GE) per mL), only 0.5% of sequencing reads produced by unbiased shotgun sequencing were derived from IFV (‘on target’ reads), whereas by stark contrast, the majority of reads produced by target enrichment sequencing (54.4%) were derived from IFV.

Given the dramatic increase in sensitivity observed when complex samples were spiked with an individual virus’s genetic material and subjected to target enrichment, we next sought to evaluate whether these effects would still be observed in the presence of an additional virus and at lower concentrations of IFV gRNA overall. Therefore, IFV gRNA was spiked into total RNA derived from Middle Eastern Respiratory Syndrome Coronavirus (MERS-CoV) cell culture lysate at an overall lower range of increasing concentrations than IFV was spiked in the prior experiment. As before, the samples were aliquoted into two parts that were processed by each method. In these synthetic co-infection samples, target enrichment sequencing resulted in a simultaneous increase in sensitivity for both viruses as compared to unbiased, shotgun sequencing (Fig. 1b). As expected, a constant high proportion of reads mapping to MERS-CoV was observed and the proportion of reads mapping to IFV increased in a dose-dependent fashion with the number of genome equivalents spiked (Additional file 2: Table S2).

### Detection and discrimination of related viruses in clinical samples

**Fig. 2:**
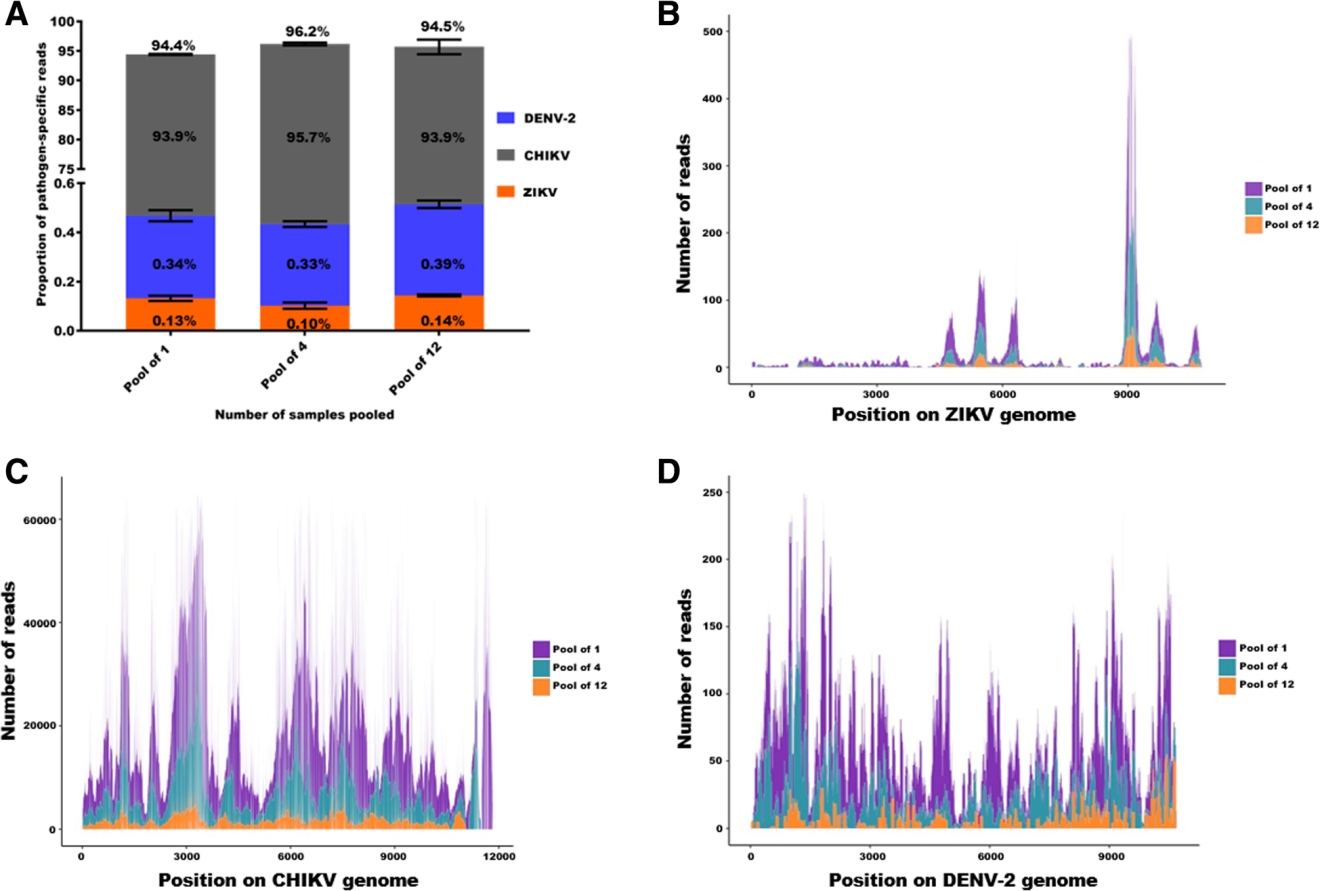
Discrimination of ZIKV, CHIKV, and DENV in mock clinical samples at clinically relevant titers. Sequencing libraries made from serum samples spiked with ZIKV, DENV-2 and CHIKV were prepared in duplicate and either probed singly or within pools of four or 12. **a** The percentage of pathogen-specific reads detected within the synthetic co-infection samples is shown, along with the standard error of the mean for the two replicates. **b**-**d** Coverage plots demonstrating the number of reads that mapped to ZIKV, CHIKV, and DENV, respectively, as well as the distribution of those reads along the length of each genome

We next tested the sensitivity for detection of three clinically-relevant viruses that can co-circulate in tropical regions, can present with similar symptoms, and can be difficult to detect at low titers [21]. Mock clinical samples were constructed containing combinations of ZIKV, CHIKV, and DENV at loads that correlate with real clinical loads from human specimens. Briefly, varying titers of ZIKV, Dengue virus 2 (DENV-2), and Chikungunya virus (CHIKV) were spiked into RNA extracted from human serum, in duplicate, to create synthetic co-infection samples. Negative control samples consisted solely of RNA extracted from human serum. Viruses were spiked-in at concentrations corresponding to Ct values from standard curves generated via RT-qPCR. The targeted spike-in values were chosen based on reports in the literature for clinical samples containing each virus to mimic a realistic co-infection scenario [22–25]. Given that in the literature there is at least one report of clinical samples being probed singly rather than pooled and probed [9] and it is not well known how pooling may affect virus detection levels, in this experiment we also sought to evaluate whether probing singly or within a pool would affect our ability to identify virus. Therefore, total RNA extracted from these samples was probed singly (“pool of 1”) and also pooled in groups of four and 12 with singly-spiked and mock-spiked serum samples consisting of the other components of the pool. The resulting sequence reads were mapped to the reference genomes for each of these three viruses. In all cases, the three co-infecting viruses were able to be detected in each sample at relatively consistent proportions regardless of the number of samples within a pool (Fig. 2a). Even DENV-2 was detectable within each sample it was spiked, despite the low concentration of viral RNA (estimated 100 genome equivalents per mL).

Although each targeted virus was represented by enough sequencing reads to be easily detected, there were differences in the depth and breadth of genome coverage observed. Whereas the full CHIKV genome was recovered from all spiked samples at a very high depth of coverage (Fig. 2c), in the case of DENV-2, an average of 92.2% of the genome was recovered from co-infected samples (Fig. 2d) and the average ZIKV linear genome coverage was lower, at 68.8% (Fig. 2b). These patterns in coverage were similar for both replicates (Additional file 3: Figure S1). Overall, the proportion of reads mapping to a given virus was consistent and reproducible regardless of whether a sample was probed singly or probed within a group of four or 12.

**Fig. 3:**
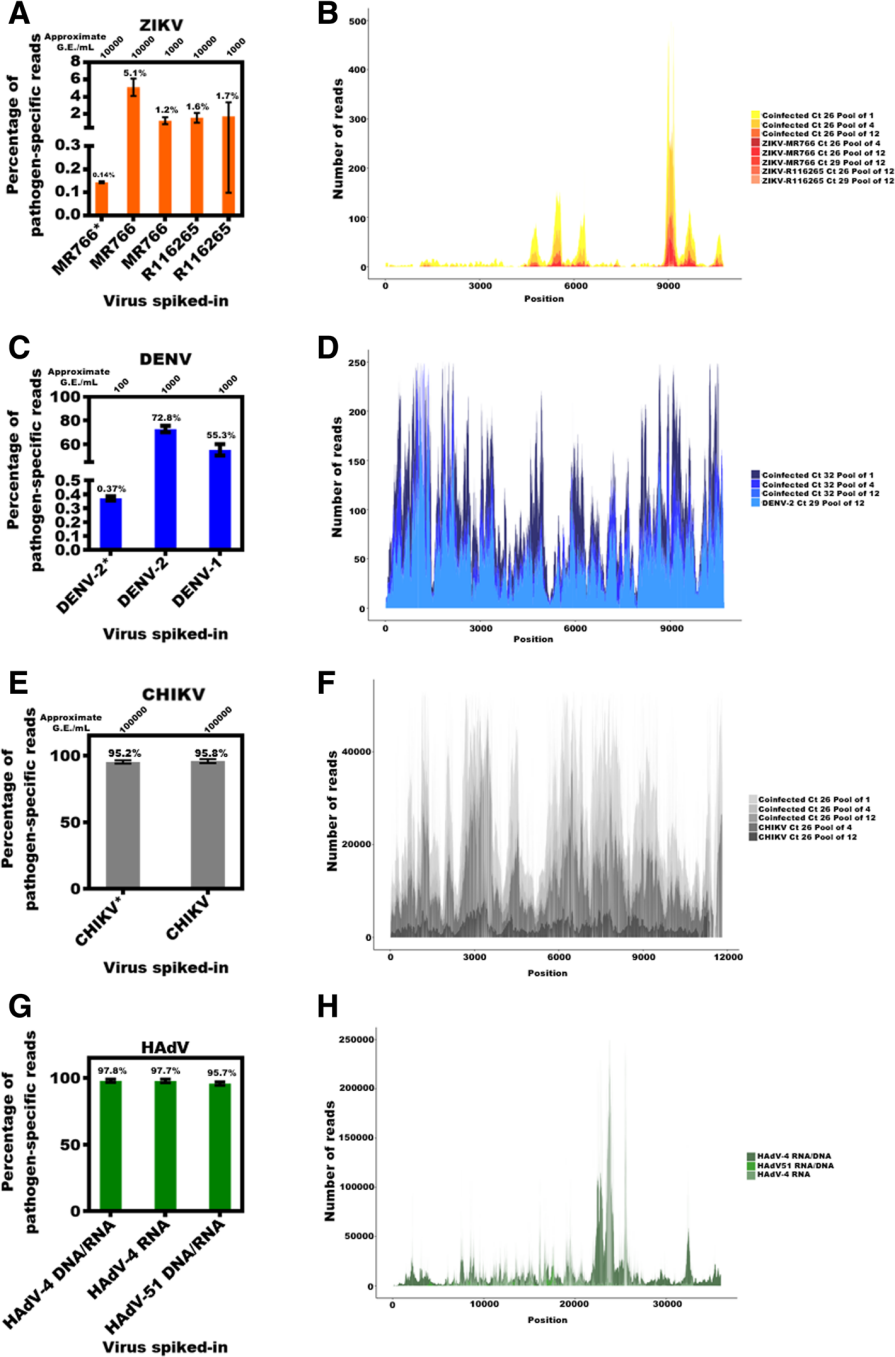
Detection of close relative viruses irrespective of extensive multiplexing. Sequencing libraries made from serum samples spiked with ZIKV, DENV, CHIKV and/or HAdV were prepared in duplicate and probed singly or probed in pools of four or 12. **a**, **c**, **e**, **g** The percentage of reads that map to each strain of spiked-in ZIKV, DENV, CHIKV, and HAdV, respectively. Each co-infected sample is denoted with an asterisk (*). Estimated genome equivalents per mL as extrapolated from RT-qPCR standard curves are listed along the top of each graph. The standard error of the mean of two replicates is shown. **b**, **d**, **f**, **h** Coverage plot for replicate one of each ZIKV-, DENV-2-, CHIKV-, and HAdV-containing sample, respectively

In addition to evaluating whether sensitivity and reproducibility are maintained despite multiplexing in pools of four and 12, we also sought to evaluate the probe panel’s performance in the context of strain-level, and even species-level, genetic variation as well as differing concentrations of viral genetic material. Specifically, synthetic clinical samples were also constructed to contain a different strain of ZIKV than the strain the probes were designed against (strain R116265 rather than strain MR766, which is the strain whose reference genome was used for probe design; Fig. 3a and b) and a different species of Human Adenovirus (HAdV) than the probes were designed to target (HAdV-51, a member of species D; as opposed to species C, B, and E, which the probes target specifically; Fig. 3g and h). These samples were also constructed to include biological replicates and were probed singly or in pools of four or 12. In all cases, the spiked-in virus was detectable, although there was some variation in depth of coverage among multiplexed samples. As might be expected, samples that were multiplexed in sets of 12 yielded the lowest depth of coverage compared to samples that were multiplexed in sets of four or probed singly (Fig. 3b, d, f). The target genomes were completely covered in the majority of on-target samples. The exception to this rich, consistent coverage included both strains of ZIKV, which, although both were detectable, did not achieve 100% linear coverage and exhibited lower depth of coverage than the other viruses that were spiked in at similar levels (Fig. 3b).

It should be noted that in this experiment, both purified RNA as well as total nucleic acid samples containing HAdV-4 and HAdV-51 genetic material were processed and sequenced (Fig. 3g and h). The total nucleic acid samples were processed without a DNase step to allow for potential detection of both the DNA viral genome as well as viral transcripts. In the case of both HAdV-4 (species E) and HAdV-51 (species D, not targeted specifically by probes), the vast majority of the resulting sequencing reads were virus-specific and the reads were well-distributed along the length of the genome in coding regions, and in the case of the total nucleic acid samples, noncoding regions as well (Fig. 3h), a phenomenon that was consistent between replicates (replicate coverage data is shown in Additional file 4: Figure S2).

### Strain-specific detection of DENV at titers below limit of detection by conventional shotgun HTS or amplicon-based sequencing

**Fig. 4:**
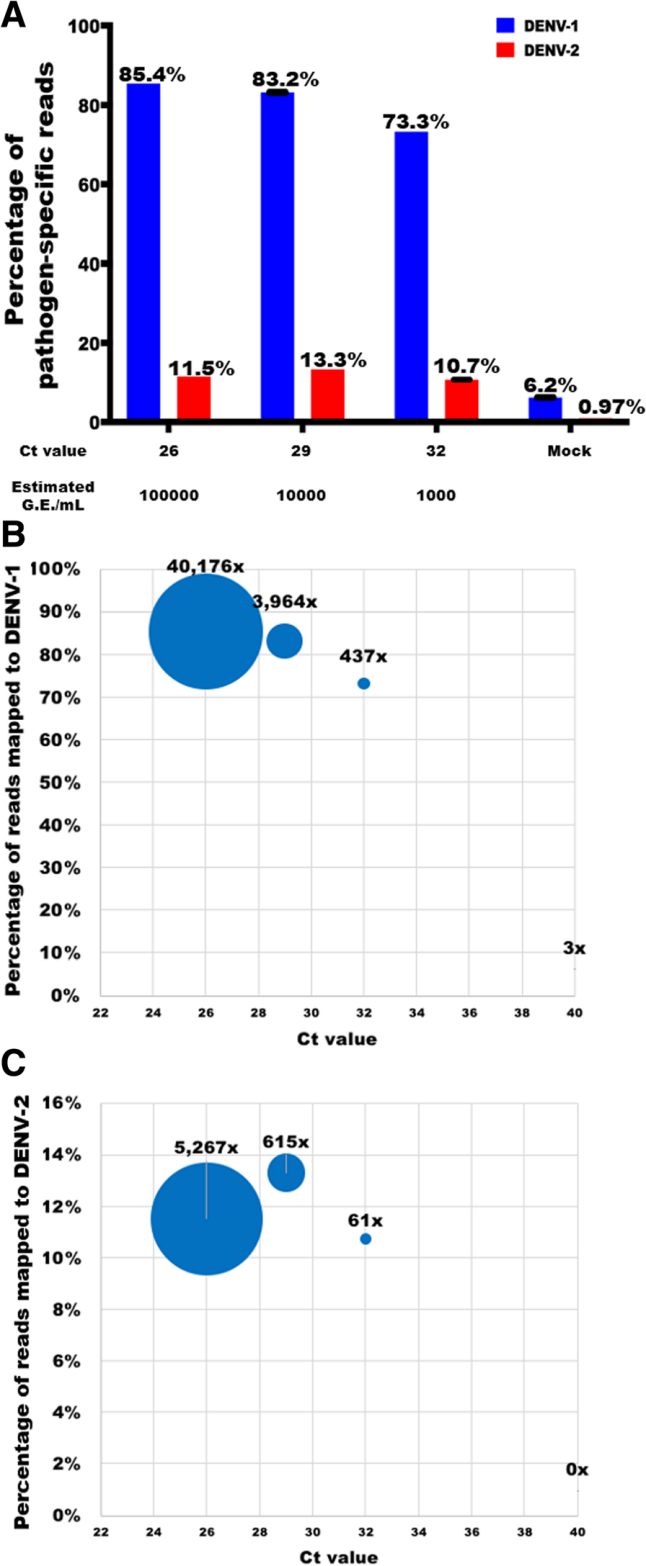
Recovery of full DENV genome at titers below limit of detection by conventional shotgun HTS or amplicon-based sequencing. DENV-1 and DENV-2 RNA were spiked into human serum RNA at a range of GE corresponding to Ct 26–32, in duplicate, and libraries were prepared using target enrichment in pools of four. Corresponding estimated genome equivalents per mL as extrapolated from a standard RT-qPCR curve are listed below the axis. Mock samples consisted of human serum RNA extract only. **a** The proportion of total reads that map to DENV-1 or DENV-2 at each Ct value. Error bars show standard error of two replicates. **b**-**c** The proportion of reads that map to DENV-1 or DENV-2, respectively, at each spike-in level. Bubble size corresponds to depth of coverage of the viral genome (average of two replicates)

It can be difficult to detect DENV-1 and DENV-2 in clinical samples when the Ct value crosses above 29 [5]. Therefore, spiked samples were created using two serotypes of DENV with Ct values corresponding to low titer, and the samples were subjected to hybridization-based enrichment and sequencing. The resulting sequence reads were found to cover the entirety of each target genome, even for the samples corresponding to Ct value 32 (estimated 1000 genome equivalents per mL). A dose-dependent response was observed in the percentage of DENV-specific reads as the Ct value decreased (Fig. 4a). For each serotype, the depth of coverage was greater than 50x even when the Ct value crossed 29 (Fig. 4b and c). As expected, the remaining reads that did not map to DENV-1 or DENV-2 were derived from human genes in spiked-in human serum RNA extract that were pulled down by the control probes, as well as the sequencing control library for PhiX.

## Discussion

A major challenge faced in virus detection as well as virus sequencing is the difficulty to detect divergent strains of viruses typically present at low titers amongst a robust host or environmental background. Small viral genomes present at low concentrations are effectively drowned out by signal from host nucleic acid and from commensal microorganisms. A variety of methods have been employed to increase the viral signal in high-throughput sequence data, including amplicon sequencing, but for viruses like DENV, with its genome of less than 11 kb in size, even amplicon sequencing is regarded as an inefficient approach for samples with Ct values of 29 or higher [5]. Such limitations have been of particular concern for U.S. Department of Defense (DoD) laboratories tasked with biosurveillance and biodefense activities in regions with limited material resources and human expertise. Part of the motivation for this effort was to provide DoD laboratories operating in austere environments new tools aimed at enhancing on-site sequencing capacity when engaged in Force Health Protection (FHP) activities.

We demonstrate here that hybridization-based viral target enrichment yields robust coverage of small genomes from clinical samples, even yielding full-length, deeply covered genomes at concentrations whereby current amplicon sequencing protocols may be expected to fail. Moreover, we demonstrate that hybridization-based target enrichment can allow for not only detection, but also genetic characterization such as strain-level discrimination, even at very low concentrations of virus. The capability to detect and discriminate between multiple serotypes of a virus within a complex sample at clinically relevant concentrations by using this enrichment method increases the utility of high throughput sequencing for biosurveillance and for infectious disease diagnostics. For both biosurveillance and clinical sequencing, assay cost and time are important considerations. We have demonstrated that more extensive pooling and multiplexing can be performed to reduce cost and time without sacrificing the assay’s ability to detect at least two strains of related virus and a variety of unrelated viruses in one sequencing reaction.

To date, the published viral target enrichment studies vary in focus and include characterization of EBOV during a recent outbreak in West Africa [9, 10] and detection of multiple viral families within clinical samples [13, 16]. While the probes employed in the studies published to date vary in length from 50- to 120-mers, enrichment methods also can differ by the number of probes and target viruses included in a set. An additional potential protocol difference is the number of samples pooled, which ranges from a single sample to 12 [9, 10, 15]. The current recommendation by Illumina for viral enrichment is to pool four samples [14].

The experiments described here systematically test enrichment of a single library as well as pools of four or 12 libraries and include a variety of titers of as many as three viruses within a single sample and as many as 12 samples within an enriched pool. For all conditions, even with more extensive pooling and multiplexing, we observed a dose-dependent response to varying Ct values even in co-infected clinical samples. A dose dependent response was also observed by O’Flaherty et al. in two co-infected samples containing Respiratory Syncytial virus and human Coronavirus OC43 spiked-in each at Ct 28 or 32 [13]. Interestingly, although there was the expected dose-dependent effect on the proportion of sequencing reads derived from IFV as the concentration of spiked IFV gRNA increased, there was a slight decrease in the proportion of MERS-CoV-derived sequencing reads in samples at the upper end of the IFV gRNA concentration range. This was not expected given that MERS-CoV was present in each replicate at a constant, high level. We hypothesize that this may be due to saturation of the streptavidin beads used to capture probe-cDNA hybridized fragments. Further experimentation will be required to test that hypothesis.

Efficacy of probes varies by homology to the viral target, as evidenced by our results and those in the literature [13, 15]. For example, the reference sequence used to design the probe set for DENV-1 exhibits 74% nucleotide identity over 35% of the length of the closest sequenced reference for the DENV-2 strain that was spiked. It is possible that this overlap, which is not shared by the DENV-2 probes and DENV-1 spike-in, contributed to an overrepresentation of DENV-1 reads in the experiments presented in Figs. 3 and 4. Additionally, by comparison to the other richly covered strains of virus tested in multiplexed samples, there was an underwhelming coverage for both strains of ZIKV. It is possible that the quality of RNA from these viruses was less than the other RNA spike-ins, or that the probe panel for ZIKV is less efficacious when used in combination with the entirety of the probe set. The probes for ZIKV were synthesized and added later after all the other probes were combined (in response to the recent outbreak) and therefore it is possible that the comparatively lower performance of the ZIKV probes is due to a difference in quantitation of the ZIKV probe set.

We observed that HAdV-51 (species D) genetic material was efficiently enriched even though the only adenoviruses used to design the probe panel were species B, C, and E. This experiment indicates that the protocol works as well for this particular DNA virus as it does for RNA viruses. Limits of detection may vary by viral target, which may explain why previously published experiments showed differences between DNA and RNA viruses [13]. Nucleotide identity between human adenovirus species D (the species to which HAdV-51 belongs) and the other human adenovirus species HAdV-A, B, C, and E was reported by Kaneko et al. to range from 58.73 to 69.35% [26]. In our study, the probe panel containing probes for species HAdV-B, C, and E effectively enriched for the entirety of the HAdV-D genome. This suggests that when using long (80-mer) probes designed against several species of virus, related non-targeted species may also be enriched without being specifically included in the panel, if the nucleotide identity among them is at least 60–70%, and if multiple related species are targeted by the probes in the panel (in this case three species). This cross-reactivity for related human pathogens could prove to be a useful feature, by allowing for enrichment of more relevant viruses without added cost spent to increase the number of probes.

Our findings demonstrate that breadth of coverage does not suffer from extensive pooling but that deeper depth of coverage is gained by limiting the number of samples pooled. Extensive pooling makes hybridization-based enrichment sequencing more economical. Viral target enrichment could be applied as an economical approach to sequencing viruses known to mutate quickly and therefore evade other assays, fastidious organisms, or complex samples of limited volume. For example, this method could be prescribed to a scenario in which multiple serotypes of a virus such as DENV are expected to be present in a sample but detection is prohibited via conventional methods such as amplicon sequencing due to low titers. Viral target enrichment designed for a broad panel of targets could also be useful to the infectious disease field by enabling detection of low-titer viruses present in clinical samples taken from patients suffering from symptoms of unknown etiology. Another applicable use of a broad probe panel could be to perform environmental sampling. The aforementioned applications often involve complex samples of limited volume, for which this method is ideal. An important caveat to this approach is that while viral target enrichment is an economical method by which to reduce background noise in a metagenomic sample, probe design requires prior knowledge of the closest-sequenced genome for each viral target. Amplicon sequencing may be the best approach for previously known samples and unbiased whole shotgun sequencing may be more appropriate for a virus-rich sample. None of these approaches obviates the use of amplification by polymerase chain reaction or the potential introduction of sequence errors, and so standard quality analyses by computational methods should always be employed. As the development and testing of probe sets for viral target enrichment expands and continues, the application of this technique could make HTS more economical for routine use in Force Health Protection activities including biosurveillance, biodefense and outbreak investigations.

## Methods

### Preparation of contrived metagenomic samples and nucleic acid extraction and quality control

IFV (H1N1) particles (A/Swine/Iowa/15/30; ATCC, Manassas, VA), MERS-CoV RNA (Jordan-N3/2012; NAMRU-3), HAdV nucleic acid extract from particles (RI-67 and Bom; ATCC, Manassas, VA), ZIKV RNA extract from particles (MR766 and R116265; ATCC, Manassas, VA), CHIKV RNA (gift from LTC Richard Jarman, Walter Reed Army Institute of Research), DENV-1 RNA extract from particles (TH-SMAN; ATCC, Manassas, VA) and DENV-2 RNA extract from particles (New Guinea C; ATCC, Manassas, VA) were spiked into relevant matrices to construct contrived metagenomic samples for testing.

To prepare guano samples five-gram quantities of commercial Jamaican bat guano (Planet Natural; Bozeman, MT) were placed in 50 mL of sterile-filtered Hank’s Balanced Salt Solution (HBSS), vortexed to mix, centrifuged at 3,100 x g for 10 min, and filtered sequentially through 0.45 μm and 0.22 μm filters prior to spiking with IFV particles. Post-addition of IFV, samples were centrifuged at 39,000 x g for three hours at 10 °C to concentrate spiked and native virus particles, supernatant was removed, and total RNA was extracted from the pellet using the QIAampViral RNA Isolation Kit (QIAGEN; Valencia, CA). After elution in 30 μL buffer AVE, a second elution using 20 μL of the eluate was performed.

To prepare cell culture matrix samples, a nucleic acid spiking approach was used. In this case, decreasing amounts of IFV RNA were spiked into a constant mass of total RNA that had been extracted from Vero cells infected with MERS-CoV. Aliquots of the same Vero cell culture were used for each sample. Genome equivalents of IFV spiked into samples were calculated based on RNA mass extracted from virus particles and genome size.

For serum samples, RNA was extracted from Human Serum (BioIVT, Westbury, NY) and mixed with viral RNA*.*

Total nucleic acid was extracted from adenovirus particles using the QIAGEN QiAMP MinElute Virus Spin Kit, omitting carrier RNA. The samples were eluted in 24 μl buffer AVE. Viral RNA was extracted from virus particles and human serum using the QIAampViral RNA Isolation Kit as described above. The Qubit double stranded DNA Broad-Range Assay Kit and the Qubit RNA Broad-Range Assay Kit (Thermo Fisher Scientific; Waltham, MA) were used to assay extracts.

### Quantitative reverse transcription PCR

For quantitative reverse transcription PCR, SuperScriptIII RT/Platinum *Taq* Mix (Thermo Fisher Scientific; Waltham, MA), dNTP mix, MgSO_4_, ROX Reference Dye (Thermo Fisher Scientific; Waltham, MA), and the primers and probes listed in Table 2 were used to assay in the CFX Connect Real-Time PCR Detection System (Bio-Rad; Hercules, CA) using the following conditions: 50 °C for 15 min, 95 °C for two minutes, and 40 cycles of 95 °C for 15 s and 60 °C for one minute. Standard curves for DENV-1, DENV-2, CHIKV and ZIKV were generated from titrated viral RNA extract.

**Table 2:** Primers and probes used for qRT-PCR

Virus	Forward Primer Sequence	Reverse Primer Sequence	Probe Sequence	Reference
CHIKV (181/Clone 25)	AGCTCCGCGTCCTTTACCA	GCCAAATTGTCCTGGTCTTCCT	Express One SYBR Green I (Life Technologies)	[30]
DENV-1 (TH-SMAN)	GACACCACACCCTTTGGACAA	CACCTGGCTGTCACCTCCAT	FAM-AGAGGGTGTTTAAAGAGAAAGTTGACACGCG-TAMRA	[31]
DENV-2 (New Guinea C)	ACAGGCTATGGCACTGTTACGAT	TGCAGCAACACCATCTCATTG	FAM-AGTGCTCTCCAAGAACGGGCCTCG-TAMRA	[32, 33]
IFV-A – HA (A/Swine/Iowa/15/30)	CCAGTCACAATAGGAGAGTG	AAACCGGCAATGGCTCCAAA	Express One SYBR Green I (Life Technologies)	[34]
ZIKV (MR766 and R116265)	AARTACACATACCARAACAAAGTGG**T**^a^	TCCRCTCCCYCTYTGGTCTTG	Express One SYBR Green I (Life Technologies)	[35]

^a^boldface T was modified from originally published R

### Library preparation, virus enrichment, and sequencing

For virus enriched sequencing, TruSeq RNA Access libraries were created as per manufacturer’s protocol (Illumina; San Diego, CA), with the following two modifications: i) rather than the standard CEX oligonucleotides that are designed for enrichment of human genes, a custom pool of oligonucleotides was used that includes probes along the entire genome length of 83 viruses (Table 1) as well as probes specific for several human house-keeping genes, and ii) in the second PCR amplification, 17 cycles were used rather than ten. Samples were probed singly or in pools of four or 12 and multiplexed for sequencing on the Illumina MiSeq platform using v3 chemistry, 2X75 bp read lengths.

For conventional HTS (shotgun) sequencing, TruSeq libraries were pooled and sequenced on the MiSeq platform using v3 chemistry, 2X300 bp read lengths. For the guano samples, these consisted of an aliquot of each of the TruSeq RNA Access libraries from the step prior to virus enrichment. For the MERS-CoV-Vero cell matrix samples, these consisted of shotgun libraries made with the TruSeq RiboZero Gold Library Preparation Kit (Illumina; San Diego, CA).

### Virus enrichment probe design

A composite panel of 80-mer DNA probes was assembled using a previously described panel for respiratory viruses [13, 14, 20], a previously described panel for Filoviruses [9–11], plus an additional panel of newly designed probes for 41 viruses of biosurveillance and biodefense concern, for a total of 19,077 probes. The methods employed for capture oligo design were essentially as described in O’Flaherty at al [13], although the design varied somewhat across the target viral genomes. For instance, in the case of the previously described respiratory virus panel, the design was focused on coding regions [13]. In general, genomes were tiled with capture oligos in a way so as to avoid low-complexity sequences and repetitive sequences. Probe spacing and overlap vary per virus due to attempts to design probes that cover multiple related virus strains resulting in overlapping tiled design around more variable regions, whereas regions more conserved among multiple strains resulted in probes more or less tiled end-to-end. All probes were biotinylated on the 5′ end. Sequences of viral capture probes are provided in Additional file 5.

### Bioinformatic analyses

Quality control, de novo assembly, taxonomic classification, and reference-based analyses were conducted using EDGE Bioinformatic software v 2.0 [27] with default parameters and host removal of human reference GRCh38 and also with CLC Genomics Workbench v11 (QIAGEN Bioinformatics; Redwood City, CA). The reference mapping parameters in CLC were modified from defalt settings to 0.8 length fraction and 0.8 similarity fraction with global alignment and random mapping of non-specific matches. BLAST [28] was also used to further investigate specific datasets. The ggplot2 R package was used to generate depth of coverage plots [29].

## Additional files

Additional file 1: **Table S1.** Number and proportion of reads mapped to IFV at spiked-in genome equivalents of 0, 1,250, 3,750 and 5,000 given preparation by hybridization-based target enrichment or shotgun sequencing. (DOCX 12 kb)

Additional file 2: **Table S2.** Number and proportion of reads mapped to IFV or MERS-CoV at IFV spiked-in genome equivalents of 0, 750, 1,500 and 3,000 and a constant, high level of MERS-CoV genomic material given preparation by hybridization-based target enrichment or shotgun sequencing. (DOCX 15 kb)

Additional file 3: **Figure S1.** Coverage plots demonstrating the number of reads that mapped to ZIKV, CHIKV, and DENV, respectively, as well as the distributionof those reads along the length of each genome. Replicates shown in Figure 2. (TIF 338 kb)

Additional file 4: **Figure S2.** Coverage plot for replicate one of each ZIKV-, DENV-2-, CHIKV-, and HAdV-containing sample, respectively. Replicates shown in Figure 3. (TIF 655 kb)

Additional file 5: Nucleotide sequences for the custom pool of oligonucleotides that includes probes along the entire genome length of 83 viruses. (XLSX 1043 kb)

## Abbreviations

bp: base-pair
CEX: Coding exome
CFU: Colony-forming units
CHIKV: Chikungunya virus
Ct: Cycle threshold
DENV: Dengue virus
DNA: Deoxyribonucleic acid
ds: Double-stranded
EBOV: Ebola virus
FFPE: Formalin-fixed paraffin-embedded
GE: Genome equivalents
gRNA: Genomic RNA
HA: Hemagglutinin
HAdV: Human Adenovirus
HTS: High-Throughput Sequencing
IFV: Influenza virus
kb: kilobase
MERS-CoV: Middle Eastern Respiratory Syndrome Coronavirus
NGS: Next-Generation Sequencing
PCR: Polymerase Chain Reaction
PFU: Plaque-forming units
RNA: Ribonucleic Acid
RT-PCR: Real-time PCR
RT-qPCR: Reverse transcription quantitative PCR
RVP: Respiratory Virus Panel
ss: Single-stranded
ZIKV: Zika virus
