# Supplementary material for: Enrichment post-library preparation enhances the sensitivity of high-throughput sequencing-based detection and characterization of viruses from complex samples
Source: BMC Genomics. 2019 Feb 26;20:155. doi: 10.1186/s12864-019-5543-2 (PMC6390631; doi:10.1186/s12864-019-5543-2)
Supplement: Supplementary file 1 — Table S1. Number and proportion of reads mapped to IFV at spiked-in genome equivalents of 0, 1,250, 3,750 and 5,000 given preparation by hybridization-based target enrichment or shotgun sequencing. (DOCX 12 kb) [file 12864_2019_5543_MOESM1_ESM.docx]

Supplemental Table S1

| Spike-in level (IFV GE) | **Enriched** | | **Shotgun** | |
| --- | --- | --- | --- | --- |
|  | Number of reads mapped to IFV (%) | Total number of reads | Number of reads mapped to IFV (%) | Total number of reads |
| 0 | 1,551 (0.1) | 950,334 | 88 (0.0) | 7,408,474 |
| 1,250 | 2,004,766 (54.4) | 3,683,304 | 31,783 (0.5) | 6,295,476 |
| 3,750 | 2,828,992 (73.9) | 3,825,462 | 160,727 (2.0) | 7,964,390 |
| 5,000 | 9,431,355 (79.4) | 11,879,278 | 161,078 (4.2) | 3,820,210 |
