# Supplementary material for: Enrichment post-library preparation enhances the sensitivity of high-throughput sequencing-based detection and characterization of viruses from complex samples
Source: BMC Genomics. 2019 Feb 26;20:155. doi: 10.1186/s12864-019-5543-2 (PMC6390631; doi:10.1186/s12864-019-5543-2)
Supplement: Supplementary file 2 — Table S2. Number and proportion of reads mapped to IFV or MERS-CoV at IFV spiked-in genome equivalents of 0, 750, 1,500 and 3,000 and a constant, high level of MERS-CoV genomic material given preparation by hybridization-based target enrichment or shotgun sequencing. (DOCX 15 kb) [file 12864_2019_5543_MOESM2_ESM.docx]

Supplemental Table S2

| Spike-in level  (IFV GE) | **Enriched** | | | | **Shotgun** | | | |
| --- | --- | --- | --- | --- | --- | --- | --- | --- |
|  | Replicate | Number of reads mapped to MERS-CoV (%) | Number of reads mapped to IFV (%) | Total number of reads | Replicate | Number of reads mapped to MERS-CoV (%) | Number of reads mapped to IFV (%) | Total number of reads |
| 0 | 1 | 2,155 (0.6) | 101 (0.0) | 387,990 | 1 | 39 (0.0) | 4 (0.0) | 1,561,344 |
|  | 2 | 174 (0.3) | 6 (0.0) | 63,176 | 2 | 371 (0.0) | 26 (0.0) | 6,064,582 |
|  | 3 | 1,144 (0.4) | 48 (0.0) | 314,438 | 3 | 326 (0.0) | 24 (0.0) | 14,594,702 |
| 750 | 1 | 15,583,878 (91.3) | 395,920 (2.3) | 17,066,356 | 1 | 357,485 (4.9) | 11,332 (0.2) | 7,365,006 |
|  | 2 | 2,827,898 (91.8) | 72,904 (2.4) | 3,079,278 | 2 | 27,662 (4.2) | 1,125 (0.2) | 653,402 |
|  | 3 | 8,424,349 (92.4) | 170,642 (1.9) | 9,117,676 | 3 | 555,541 (4.7) | 19,697 (0.2) | 11,806,002 |
| 1,500 | 1 | 4,242,322 (89.4) | 223,553 (4.7) | 4,745,628 | 1 | 489,512 (4.1) | 33,170 (0.3) | 11,978,122 |
|  | 2 | 2,128,591 (89.4) | 111,979 (4.7) | 2,381,744 | 2 | 330,164 (4.7) | 25,037 (0.4) | 7,001,470 |
|  | 3 | 9,412,289 (88.5) | 447,424 (4.2) | 10,628,330 | 3 | 589,841 (4.1) | 47,253 (0.3) | 14,386,164 |
| 3,000 | 1 | 4,639,964 (85.4) | 444,109 (8.2) | 5,431,254 | 1 | 59,736 (3.5) | 7,429 (0.4) | 1,700,186 |
|  | 2 | 8,963 (26.3) | 801 (2.4) | 34,048 | 2 | 236,784 (4.9) | 28,929 (0.6) | 4,876,586 |
|  | 3 | 7,762,432 (85.0) | 835,581 (9.2) | 9,135,164 | 3 | 474,837 (3.5) | 79,902 (0.6) | 13,480,476 |
