# Supplementary figures and images for: Enrichment post-library preparation enhances the sensitivity of high-throughput sequencing-based detection and characterization of viruses from complex samples
Source: BMC Genomics. 2019 Feb 26;20:155. doi: 10.1186/s12864-019-5543-2 (PMC6390631; doi:10.1186/s12864-019-5543-2)

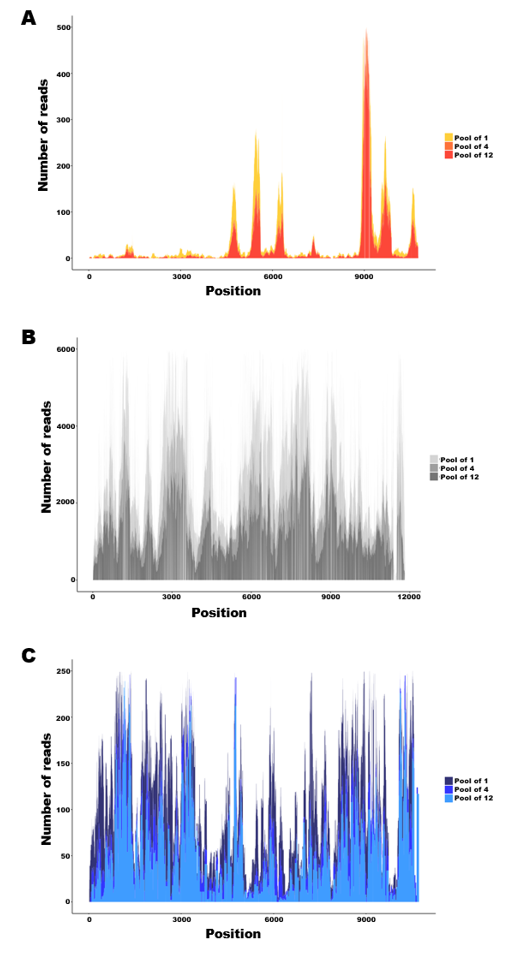

Supplement: Supplementary file 3 — Figure S1. Coverage plots demonstrating the number of reads that mapped to ZIKV, CHIKV, and DENV, respectively, as well as the distributionof those reads along the length of each genome. Replicates shown in Figure 2. (TIF 338 kb) [file 12864_2019_5543_MOESM3_ESM.tif]

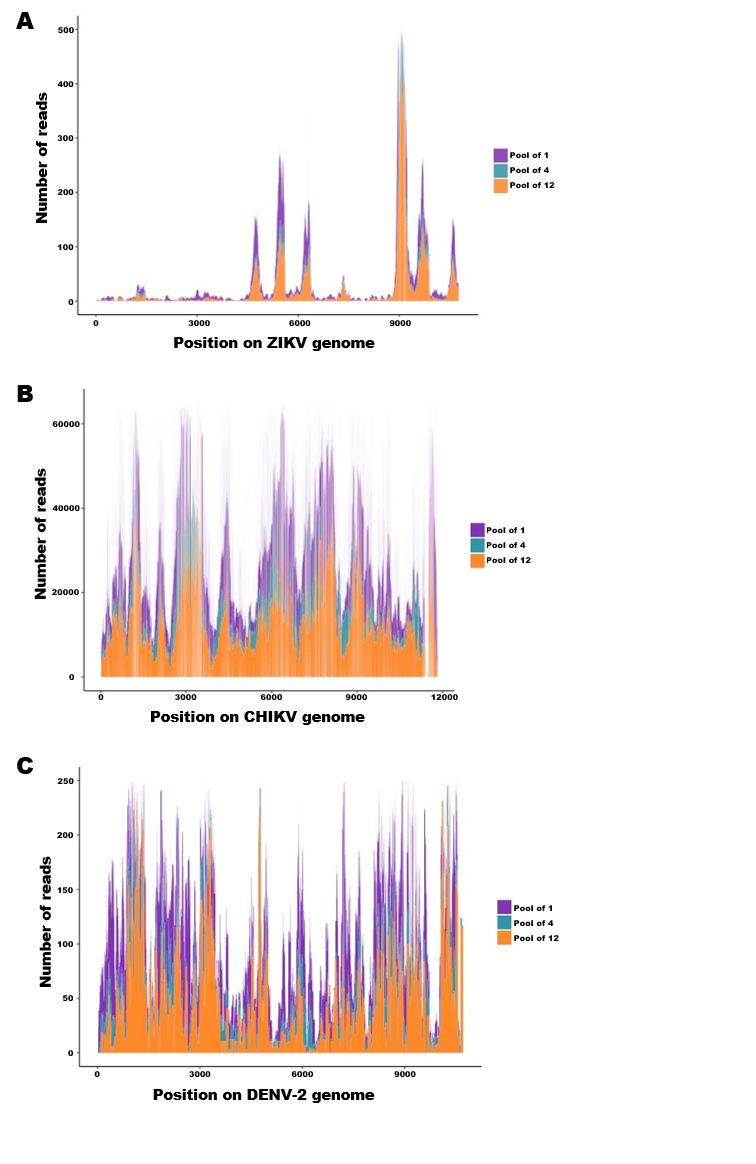

Supplement: Supplementary file 4 — Figure S2. Coverage plot for replicate one of each ZIKV-, DENV-2-, CHIKV-, and HAdV-containing sample, respectively. Replicates shown in Figure 3. (TIF 655 kb) [file 12864_2019_5543_MOESM4_ESM.tif]
